# Unitarity constraints on general scalar couplings with SARAH

**DOI:** 10.1140/epjc/s10052-018-6127-z

**Published:** 2018-08-14

**Authors:** Mark D. Goodsell, Florian Staub

**Affiliations:** 1 Laboratoire de Physique Théorique et Hautes Energies (LPTHE), UMR 7589, Sorbonne Université et CNRS, 4 place Jussieu, 75252 Paris Cedex 05, France; 2 Institute for Theoretical Physics (ITP), Karlsruhe Institute of Technology, Engesserstraße 7, 76128 Karlsruhe, Germany; 3 Institute for Nuclear Physics (IKP), Karlsruhe Institute of Technology, Hermann-von-Helmholtz-Platz 1, 76344 Eggenstein-Leopoldshafen, Germany

## Abstract

We present an update of the Mathematica package SARAH to calculate unitarity constraints in BSM models. The new functions can perform an analytical and numerical calculation of the two-particle scattering matrix of (uncoloured) scalars. We do not make use of the simplifying assumption of a very large scattering energy, but include all contributions which could become important at small energies above the weak scale. This allows us to constrain trilinear scalar couplings. However, it can also modify (weakening or strengthening) the constraints on quartic couplings, which we show via the example of a singlet extended Standard Model.

## Introduction

In a classic paper, Lee, Quigg and Thacker showed that the Higgs mass in the Standard Model (SM) must be below 1 TeV in order to maintain perturbative unitarity [1]. From the measurement of the Higgs mass at the LHC [2, 3], we have learned that the quartic coupling in the SM is even well below 1, i.e. the scalar sector of the SM has very weak self-couplings. However, this is not necessarily true if one adds more fundamental scalars to the theory. The scalar potential of BSM models often involve many new parameters which are experimentally barely constrained. Therefore, theoretical conditions like the stability of the potential or the conservation of unitarity are very important to find physical viable parameter regions in these models.

The constraints from tree-level perturbativity are often applied in well studied models such as ones with additional singlets [4–6], doublets [7–13], or triplets [14–16]. However, very often the constraints are derived under the assumption that the scattering energy is much larger than the involved masses. In this limit, only point interactions are important, and all diagrams with propagators are neglected. As a consequence, cubic couplings do not enter the widely used constraints at all. However, it was already pointed out long ago that large cubic couplings of light scalars are dangerous [17], and also limits for the triple Higgs coupling in the SM were derived [18, 19]. In this work, we shall present a general calculation of unitarity constraints without the assumption of a very large scattering energy, with the following motivation:

1. We want to place bounds on genuine trilinear couplings.
2. For theories where additional scalars couple to the Higgs, even if there are no trilinear couplings before electroweak symmetry breaking, they are generated after the Higgs takes a vev, and unitarity of scattering at finite *s* gives new constraints on these quartic couplings.
3. For theories defined with a low cutoff, scattering may never be in the regime where the energies are sufficiently large to neglect the *s*, *t*, *u*–channel processes.
4. Even for theories with a high cutoff, the infinite energy approximation is rarely justified since the couplings must run: if we take the energy sufficiently high to be able to neglect particle masses, the resummed couplings will typically have completely different values.

For this purpose we have extended the Mathematica package SARAH [20–24] by routines for the analytical and numerical study of the full tree-level unitarity constraints. While the analytical routines are helpful to obtain expressions for $2\rightarrow 2$ scattering elements, a symbolic calculation of the full scalar scattering matrix could become slow and less illuminating. Therefore, for practical application the Fortran output for SPheno [25, 26] has been also extended to obtain a numerical prediction for the maximal eigenvalue of the full scattering matrix.

We discuss in sect. 2 the underlying calculations to obtain unitarity constraints in generic BSM models, and the assumptions/restrictions that we shall apply. The importance of the full calculation is demonstrated in sect. 3 via the example of singlet extensions of the SM. In sect. 4 we show how the new routines are used. A brief summary is given in sect. 5.

## Generic calculation of unitarity constraints

### $2\rightarrow 2$ Scattering processes of scalars at finite momentum

The derivation of unitarity constraints is elementary, but the derivation for finite momentum is rarely found in the literature – and there are many common misunderstandings – so we present a clear exposition in Appendix A. The result is that the partial wave constraint becomes

1 $$\begin{aligned} -i (a_J - a_J^\dagger ) \le a_J a_J^\dagger \qquad \forall J \end{aligned}$$

where $a_J$ is a normal matrix related to the partial wave decomposition of $2 \rightarrow 2$ scattering matrix elements $\mathcal {M}_{ba}$ from a scattering of a pair of particles $a= \{1,2\}$ with momenta $\{p_1, p_2\}$ to a pair $b = \{3,4\}$ with momenta $\{k_3, k_4\}$ as

2 $$\begin{aligned} a_J^{ba} \equiv&\frac{1}{32\pi } \sqrt{\frac{4 |\mathbf {p}^b| |\mathbf {p}^a|}{2^{\delta _{12}} 2^{\delta _{34}}\, s}} \int _{-1}^1 d(\cos \theta ) \mathcal {M}_{ba} (\cos \theta ) P_J (\cos \theta ). \end{aligned}$$

The factor $\delta _{12} (\delta _{34})$ is 1 if particles $\{1,2\} (\{3,4\})$ are identical, and zero otherwise. $P_J$ are the Legendre polynomials, $\mathbf {p}^i$ is the centre of mass three-momentum for particle *i*, and $s=(p_1 + p_2)^2$ is the standard Mandelstam variable.

From the fundamental equation (1) different constraints can be derived; we shall only consider the zeroth partial wave, and denoting $a_0^i$ as the eigenvalues of $a_0$ we shall apply

3 $$\begin{aligned} \mathrm {Re} (a_0^i) \le&\frac{1}{2}\ \forall \ i. \end{aligned}$$

**Fig. 1:**
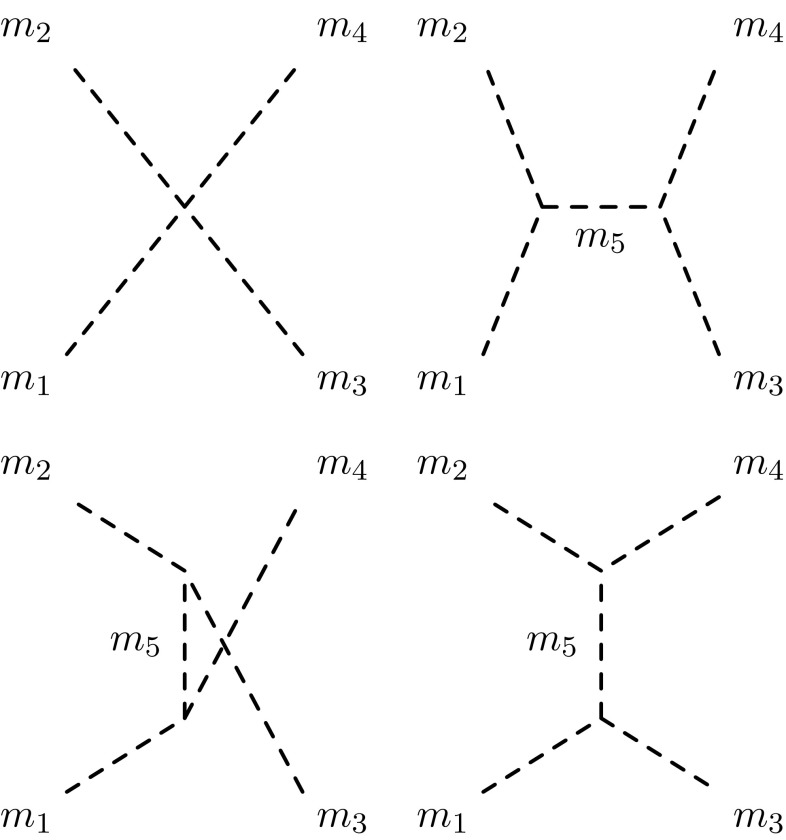
The four topologies contributing to $2 \rightarrow 2$ scalar scattering processes at finite $\sqrt{s}$. In the approximation of $\sqrt{s} \gg m_i$, only the point interaction contributes

The diagrams which contribute to $2 \rightarrow 2$ scalar scattering processes are shown in Fig. 1. For a general field theory consisting of real scalars $\phi _i$ and couplings

4 $$\begin{aligned} \mathcal {L} \supset - \frac{1}{6} \kappa ^{ijk} \phi _i \phi _j \phi _k - \frac{1}{24} \lambda ^{ijkl} \phi _i \phi _j \phi _k \phi _l \end{aligned}$$

the matrix elements are

5 $$\begin{aligned} \mathcal {M} (1,2 \rightarrow 3,4) =&- \lambda ^{1234} -\kappa ^{12 5} \kappa ^{345} \frac{1}{s - m_5^2} \nonumber \\&-\kappa ^{13 5} \kappa ^{245} \frac{1}{t - m_5^2}- \kappa ^{14 5} \kappa ^{235} \frac{1}{u - m_5^2}. \end{aligned}$$

The integration over $\cos \theta$ is trivial for the contact and *s*-channel processes, and always straightforward for the others using

6 $$\begin{aligned} t =&m_1^2 + m_3^2 - 2 E_1 E_3 + 2 |\mathbf {p}_1| |\mathbf {p}_3| \cos \theta \nonumber \\ u =&m_1^2 + m_4^2 - 2 E_1 E_4 - 2 |\mathbf {p}_1| |\mathbf {p}_3| \cos \theta , \end{aligned}$$

where $E_i$ are the energies of the particles in the centre of mass frame, and $\mathbf {p}_1, \mathbf {p}_3$ are the three-momenta. We shall express the results in terms of the function

7 $$\begin{aligned}&\lambda (s, m_i^2, m_j^2)\nonumber \\&\quad \equiv \frac{1}{s^2} \bigg [s^2 + m_i^4 + m_j^4 - 2 m_i^2 m_j^2 - 2 s m_i^2 - 2 s m_j^2\bigg ], \end{aligned}$$

so that

8 $$\begin{aligned} |\mathbf {p}_1| =&\frac{1}{2} \sqrt{s\lambda (s, m_1^2, m_2^2)}, \qquad |\mathbf {p}_3| = |\mathbf {p}_4|=\frac{1}{2} \sqrt{s\lambda (s, m_3^2, m_4^2)} \nonumber \\ E_1 =&\frac{s + m_1^2 - m_2^2}{2 \sqrt{s}}, \qquad E_3 = \frac{s + m_3^2 - m_4^2}{2 \sqrt{s}} \end{aligned}$$

allowing us to define

9 $$\begin{aligned}&f_t (s, m_1^2, m_2^2, m_3^2, m_4^2,m_5^2) \nonumber \\&\quad \equiv \frac{1}{2} \sqrt{\frac{4 |\mathbf {p}_1| |\mathbf {p}_3|}{s}} \int _{-1}^1 \text {d}\cos \theta \frac{1}{t-m_5^2} \nonumber \\&\quad = \frac{1}{s} \frac{1}{[\lambda (s, m_1^2, m_2^2) \lambda (s, m_3^2, m_4^2)]^{1/4}} \nonumber \\&\qquad \log \left( \frac{m_1^2 + m_3^2 - m_5^2 - 2 E_1 E_3 + 2 |\mathbf {p}_1| |\mathbf {p}_3|}{m_1^2 + m_3^2 - m_5^2 - 2 E_1 E_3 -2 |\mathbf {p}_1| |\mathbf {p}_3|}\right) \nonumber \\&f_u (s, m_1^2, m_2^2, m_3^2, m_4^2,m_5^2) \nonumber \\&\quad \equiv f_t(s, m_1^2, m_2^2, m_4^2, m_3^2,m_5^2). \end{aligned}$$

In terms of these, the (modified) zeroth partial waves are

10 $$\begin{aligned} a_0 =&- \frac{2^{-\frac{1}{2}(\delta _{12}+ \delta _{34})}}{16 \pi } \bigg \{ \bigg [\lambda (s, m_1^2, m_2^2) \lambda (s, m_3^2, m_4^2)\bigg ]^{1/4} \bigg [ \lambda ^{1234} \nonumber \\&+ \kappa ^{12 5} \kappa ^{345} \frac{1}{s - m_5^2}\bigg ] \nonumber \\&- \kappa ^{13 5} \kappa ^{245} f_t (s, m_1^2,m_2^2, m_3^2, m_4^2,m_5^2) \nonumber \\&- \kappa ^{14 5} \kappa ^{235} f_u (s, m_1^2,m_2^2, m_3^2, m_4^2,m_5^2)\bigg \}. \end{aligned}$$

### Handling of poles

In the neighbourhood of poles, the tree-level amplitude diverges which signals that we need to take higher-order corrections into account (which will effectively modify the divergent propagator to include the width, cutting off the divergence). Moreover, since we are only calculating unitarity constraints at tree-level, in the presence of large couplings large quantum corrections to masses may mean that the physical location of the poles is a long way away from the tree-level mass parameters. Both of these issues imply that we should not trust our results in such cases. We therefore apply the following conditions:

1. *s* **-Channel poles** Obviously, *s*-channel poles are present if any propagator mass is close to $\sqrt{s}$. In order to cut out this region, we set the *entire irreducible scattering matrix* to zero if the condition
  11 $$\begin{aligned} \left| 1 - \frac{s}{m^2}\right| > C_S \end{aligned}$$
  is violated. By default, a value of 0.25 is used for the cut variable $C_S$. $C_S$ can also be changed by the user. This condition is simpler than the one proposed in Ref. [27] which calculates the width $\varGamma$ of a particle, and puts the condition $|\sqrt{s}-m|> a m \varGamma (Q=b m)$ using suitable coefficients $a,b \gtrsim 1$. However, the impact on the scattering amplitude is expected to be small. Only for models with scalars which have a very large it might be necessary to adjust $C_S$.
2. *t* **-/** *u* **-Channel poles** Particles in the *t* and *u* channels can become on-shell. For a *t*-channel diagram, this can happen if
  12 $$\begin{aligned} m_1> m_3 + m_5 \vee m_3 > m_2 + m_5 \end{aligned}$$
  holds. Similar conditions exist, for $1 \leftrightarrow 2$ and $3 \leftrightarrow 4$. The conditions for *u*-channels are obtained by exchanging $3 \leftrightarrow 4$. These conditions (used in [27]) are only necessary to have a pole, but not sufficient – they are too conservative. In fact, the presence of such a pole also demands that the scattering energy *s* is smaller than a given value. The general conditions for the minimal scattering energy $s_\mathrm{min}$ to avoid poles are
  13 $$\begin{aligned}&s_\mathrm{min,t} \nonumber \\&\quad = \frac{1}{2 m_5} \Big (\sqrt{m_1^2-2 m_1 (m_3+m_5)+(m_3-m_5)^2} \nonumber \\&\qquad \sqrt{m_2^2-2 m_2 (m_4+m_5)+(m_4-m_5)^2} \nonumber \\&\qquad +m_1 (-m_2+m_4+m_5)+m_2 m_3+m_2 m_5\nonumber \\&\qquad -m_3 m_4+m_3 m_5+m_4 m_5-m_5^2 \Big ) \end{aligned}$$
  14 $$\begin{aligned}&s_\mathrm{min,u} \nonumber \\&\quad = \frac{1}{2 m_5} \Big (\sqrt{m_1^2-2 m_1 (m_4+m_5)+(m_4-m_5)^2} \nonumber \\&\qquad \sqrt{m_2^2-2 m_2 (m_3+m_5)+(m_3-m_5)^2} \nonumber \\&\qquad +m_1 (-m_2+m_3+m_5)+m_2 m_4\nonumber \\&\qquad +m_2 m_5-m_3 m_4+m_3 m_5+m_4 m_5-m_5^2 \Big ) \end{aligned}$$
  From this we find that for an often appearing, kinematic configuration with $m_3=m_1$ and $m_4 = m_2$, a *t*-channel pole only shows up for
  15 $$\begin{aligned} s < m_1 + m_2 + \frac{1}{2} \left( -m_5 + \sqrt{(-4 m_1 + m_5)(-4 m_2 + m_5)}\right) \end{aligned}$$
  We will include three different treatments of such poles, which the user can select depending on taste:
  - Only the matrix element for which such a poles appears is set to zero, but all other entries of the scattering matrix are kept. This gives the most aggressive limits.
  - A partial diagonalisation of the scattering matrix is performed as proposed in Ref. [27]: assume that *X* is the set of all kinematically accesible states at a given energy $\sqrt{s}$ and *Y* is the subset which involve states that suffer from a *t* or *u* channel pole. In that case, one can diagonalise the scattering matrix for the set $X\backslash Y$ with a unitarity matrix *U* to obtain the eigenvalues $a_0^{i,X\backslash Y}$. The condition equation (3) is changed to
    16 $$\begin{aligned} \sqrt{\mathrm {Re}(a_0^{i,X\backslash Y})^2 + R_i^2} < \frac{1}{2} \end{aligned}$$
    with
    17 $$\begin{aligned} R_i = \sum _{h \in Y}|(a_{J,Y \times X \backslash Y} U^{-1})_{ih}|^2 \end{aligned}$$
  - The entire irreducible scattering matrix is set to zero. This gives the weakest limits.

### The role of the Goldstone boson equivalence theorem

To apply unitarity constraints, in principle we should consider all coupled channels for all particles. However, in practice there are a large number available, most of which will not contribute in a meaningful way to constraints, and so in the interest of computational speed it is necessary to impose some simplifying assumptions. These are:

1. We can neglect all contributions proportional to gauge couplings, and scatter at energies well above the mass of any gauge bosons; clearly for smaller scattering energies this would mean we would be in the neighbourhood of an abundance of poles. Furthermore, light bosons mediate infra-red unsafe scattering, so our above formalism would require modification, and it is therefore reasonable to eliminate them.
2. We neglect all fermionic contributions. The above assumption partly justifies this, as the contributions to scattering from Standard Model fermions should be small at energies well above their masses.
3. To avoid an abundance of group structures of the scattering pairs, we do not consider any particles that transform under any unbroken symmetries except for the electric charge. In particular, this excludes any strongly coupled particles (such as top partners).

Assumption (1) is the most reasonable, and also most powerful: since amplitudes involving transverse gauge bosons are always proportional to gauge couplings, we can neglect them. For longitudinal gauge bosons of mass $m_V$, whose polarisation vectors can be taken to be

$$\begin{aligned} \epsilon ^\mu = \frac{1}{m_V} \left( |\mathbf {p}|, E \frac{\mathbf {p}}{|\mathbf {p}|}\right) , \end{aligned}$$

the scattering amplitudes contain factors of $1/m_V$ and hence inverse powers of the gauge couplings, so that they can have a finite amplitude as the gauge couplings are taken to zero. On the other hand, since we scatter at energies well above their masses, the Goldstone Boson equivalence theorem allows us to instead replace all external longitudinal gauge bosons with the Goldstone boson, with the important proviso that it has a physical mass equal to the gauge boson mass (so not equal to $\xi m_V$). It then turns out that, since we neglect contributions proportional to the gauge couplings, we can also neglect gauge boson propagators – but only if we work in Feynman gauge; we discuss in appendix B why this is so and what happens in other gauges.

Taken together, then, the above assumptions, and working in Feynman gauge, enable us to consider scattering amplitudes where all states are scalars. While it would be an interesting if time-consuming task to relax some of these assumptions (which we leave to future work), they are already very powerful and allow us to study a wide range of theories.

## Examples: singlet extentions of the Standard Model

We want to demonstrate the importance of the unitarity constraints beyond the large *s* approximation by a brief example.

### Pure singlet model

First we shall consider the simplest possible BSM model: the SM extended by a real singlet *S*. To illustrate point (1) in the introduction, if we just consider the singlet and assume that its couplings to the Higgs sector are small relative to its self-couplings, we can take the Lagrangian:

18 $$\begin{aligned} \mathcal {L} \supset&-\frac{1}{2} m_S^2 S^2 - \frac{1}{3} \kappa S^3 - \frac{1}{2}\lambda _S S^4 . \end{aligned}$$

There are two additional minima away from the origin if

$$\begin{aligned} \kappa ^2 > 8 m_S^2 \lambda _S, \end{aligned}$$

but the origin remains the true minimum if

19 $$\begin{aligned} \kappa ^2< 12 m_S^2 \lambda _S \pm \kappa \sqrt{ \kappa ^2 - 8 m_S^2 \lambda _S} \longrightarrow |\kappa /m_S| < 3 \sqrt{\lambda _S}. \end{aligned}$$

As a probe of genuine trilinear couplings, taking the minimum at the origin is most interesting, because once the singlet obtains an expectation value, stability constraints the trilinears to be rather small compared to the physical mass.

**Fig. 2:**
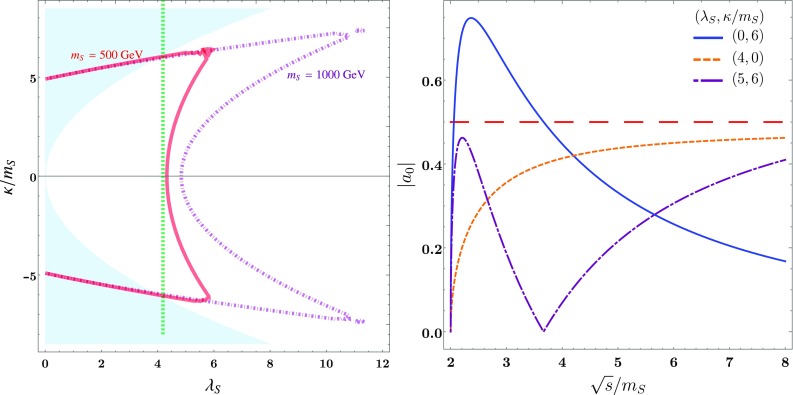
Unitarity constraints on the pure singlet model (18). Left: the solid red and dashed purple lines correspond to $m_S = 500, 1000$ GeV respectively (or other arbitrary units relative to $\sqrt{s} \le 4000$ GeV). The blue shaded regions are excluded by stability of the vacuum. Right: $|a_0|$ vs $\sqrt{s}/m_S$ for different values of the couplings $\lambda _S, \kappa /m_S$

It is simple to derive $a_0$ for this case:

20 $$\begin{aligned} a_0 =&- \frac{1}{32\pi } \bigg [\sqrt{1- \frac{4m_S^2}{s}} \bigg ( 12 \lambda _S + \frac{4 \kappa ^2}{s-m_S^2}\bigg ) \nonumber \\&\qquad + \frac{8 \kappa ^2}{\sqrt{s(s -4 m_S^2)}} \log \frac{ m_S^2}{s - 3m_S^2} \bigg ]. \end{aligned}$$

We show the constraints on this for $\sqrt{s} = 4000$ GeV, $m_S = 500$ and 1000 GeV (or other arbitrary units) in Fig. 2. Clearly the constraint from $s \rightarrow \infty$ would give $\lambda _S < \frac{4\pi }{3},$ and this is shown as the green vertical dashed line of the left-hand plot. To understand the role of the scattering energy, we show the behaviour of $|a_0|$ as $\sqrt{s}$ is varied above threshold on the right-hand plot: there is always a rapid increase followed by logarithmic behaviour.

The finite *s* constraints clearly consist of two different regimes that intersect, and come from the fact that the *t* / *u* channel contribution has opposite sign to the *s*-channel and quartic term. As a function of *s*, $$a_0$$ grows sharply from 0 at $$s = 4 m_S^2$$, before decreasing again and tending to the large *s* value. So the constraints come both from the maximum allowed *s*, and around $$s = 6 m_S^2$$; for $$\lambda _S = 0$$ it occurs at $$s \simeq 5.6 m_S^2$$. From the maximum *s*, we obtain the curved regions in the plot that have a minimum value for $$\lambda _S$$ as it passes through $$\kappa =0$$. These therefore show a difference when we change $$m_S$$. On the other hand, the overlapping curves that pass into the unstable region $$|\kappa /m_S| > 3 \sqrt{\lambda _S}$$ come from taking *s* near $$6 m_S^2$$. Note that the value $$ s = 5.6 m_S^2$$ is not near any pole value, and the corrections to the singlet mass are well under control; at one loop they are

21 $$\begin{aligned} \delta m_S^2 =\frac{2 \kappa ^2}{16\pi ^2} B_0 (p^2, m_S^2, m_S^2) + \frac{6\lambda _S}{16\pi ^2} A_0 (m_S^2), \end{aligned}$$

so for $\kappa = 5 m_S$, we have $\delta m_S^2 \sim 0.3 m_S^2$. Moreover, the scattering energy is sufficiently large that the produced particles are relativistic, so we are not in a regime where e.g. Sommerfeld enhancements would play a significant role. Hence the enhancement to the partial wave amplitude is an effect that we can use to constrain the couplings of the theory. In particular, it gives an upper bound on $\kappa$ for which trustworthy results are calculable, independent of vacuum stability considerations – especially for larger values of $\lambda _S$. We expect this to be a general feature: from an inspection of the right-hand plot of Fig. 2 we see that the strongest limits (away from poles) to a given model will either come from near-threshold production or at large *s*.

This model also allows us to simply illustrate our points (3) and (4) in the introduction. What we are interested in constraining are the values of $\kappa /m_S$ and $\lambda _S$ at low energies. However, the partial waves receive quantum corrections which can be very significant for large couplings, and if the scattering energy is large, we should certainly resum the logarithms and place constraints only on couplings evaluated $\underline{\hbox {at a renormalisation scale of }\sqrt{s}}$ (see e.g. [28]). In this model, the one-loop $\beta$-function for the quartic coupling gives

22 $$\begin{aligned} \frac{d \lambda _S}{d \log \mu }&=\frac{36 \lambda _S^2}{16 \pi ^2}, \end{aligned}$$

which can be solved exactly, and gives a Landau pole at

23 $$\begin{aligned} \mu =\,&m_S \exp \left[ \frac{4\pi ^2}{9 \lambda _S (m_S)} \right] . \end{aligned}$$

For $\lambda _S (500\ \mathrm {GeV}) = 4,$ this is at 1500 GeV! Hence we cannot apply the infinite-energy scattering limit to this coupling. Put another way, since we must understand the limits in Fig. 2 to be evaluated at $\mu = \sqrt{s}$, if $\lambda _S^\mathrm{max} (4000\ \mathrm {GeV}) = 4$, then $\lambda _S^\mathrm{max} (500\ \mathrm {GeV}) = 1.4.$

### Singlet extended SM with conserved $Z_2$

While the above model is trivial, it contains most of the ingredients that we find in more complicated models, in particular the partial cancellation between the channels. Now we will turn to a more physical example: a singlet that couples to the Higgs, but with a $Z_2$ symmetry which stabilises it and prevents mixing with the Higgs. The potential reads

24 $$\begin{aligned} V = \frac{1}{2} \lambda _H |H|^4 + \frac{1}{2} \lambda _{HS} |H|^2 S^2 + \frac{1}{2} \lambda _S S^4 \,+\, m_H^2 |H|^2 + \frac{1}{2} m_S^2 S^2 \end{aligned}$$

This theory contains no trilinear scalar couplings before electroweak symmetry breaking. As such, it is a useful prototype of popular extensions of the SM such as the Two Higgs Doublet Model, NMSSM, etc, as well as being phenomenologically interesting in its own right (for example, it provides a dark matter candidate). However, once the Higgs obains an expectation value so that we can write the neutral Higgs boson $H^0 = \frac{1}{\sqrt{2}} (v + h + i G)$, a trilinear coupling

$$\begin{aligned} \mathcal {L}\supset - \frac{1}{2} v \lambda _{HS} h S^2 \end{aligned}$$

is generated. Thus we will have *s*, *t*, *u*-channel scattering processes in the scalar sector which will modify the unitarity constraints!

In the large *s* limit we have

25 $$\begin{aligned}&\text {Max}\left\{ \left| \lambda _{HS}\right| ,\left| \lambda _H \right| ,\frac{1}{2} \left| 6 \lambda _{S}+3 \lambda _H\right. \right. \nonumber \\&\quad \left. \left. \pm \sqrt{4 \lambda _{HS}^2+36 \lambda _{S}^2+9 \lambda _H^2-36 \lambda _{S} \lambda _H }\right| \right\} <8 \pi \end{aligned}$$

We want to compare this with the full calculation. Results for the scattering processes are already given in literature [4, 5], but we disagree with both references in different channels. Therefore, we list all matrix elements in appendix C. In the following, analytical discussion, we concentrate only on the parts involving CP-even states. The scattering matrix involving only the the Higgs and the singlet is

26 $$\begin{aligned} \begin{pmatrix} hh\rightarrow hh &{} hh \rightarrow SS &{} 0 \\ SS \rightarrow hh &{} SS \rightarrow SS &{} 0 \\ 0 &{} 0 &{} hS\rightarrow hS \end{pmatrix} \end{aligned}$$

If we assume for the moment $\lambda _{HS} \gg \lambda , \lambda _S$, the dominant contribution is the $hS\rightarrow hS$ scattering.

The result reads

27 $$\begin{aligned}&16\pi a_0(hS\rightarrow hS)\nonumber \\&\quad =-\frac{\lambda _{HS}}{16 \pi s \left( s-m_S^2\right) \sqrt{m_h^4-2 m_h^2 \left( m_S^2+s\right) +\left( m_S^2-s\right) ^2}} \nonumber \\&\qquad \times \Bigg [-\left( m_h^4-2 m_h^2 \left( m_S^2+s\right) +\left( m_S^2-s\right) ^2\right) \left( -\lambda _{HS} v^2\right. \nonumber \\&\qquad \left. +m_S^2-s\right) \nonumber \\&\qquad +\lambda _{HS} s v^2 \left( s-m_S^2\right) \log \left( \frac{m_h^4-2 m_h^2 m_S^2+m_S^4-m_S^2 s}{s \left( 2 m_h^2+m_S^2-s\right) }\right) \nonumber \\&\qquad +3 m_h^2 s \left( s-m_S^2\right) \nonumber \\&\qquad \quad \log \left( \frac{m_h^2 s}{m_h^4-m_h^2 \left( 2 m_S^2+s\right) +\left( m_S^2-s\right) ^2}\right) \Bigg ] \end{aligned}$$

In order to simplify this expression we consider the limit of small $m_S$ and large $v^2\lambda _{HS}^2 \gg m_h^2 \gg m_S^2$. This results in

28 $$\begin{aligned}&16\pi a_0(hS\rightarrow hS)\nonumber \\&\quad \simeq -\frac{\lambda _{HS}^2 v^2}{s^2 \left( s-m_h^2\right) } \left( \left( m_h^2-s\right) ^2\right. \nonumber \\&\qquad \left. +s^2 \log \left( \frac{m_h^4-2 m_h^2 m_S^2-m_S^2 s}{2 m_h^2 s-s^2}\right) \right) \end{aligned}$$

**Fig. 3:**
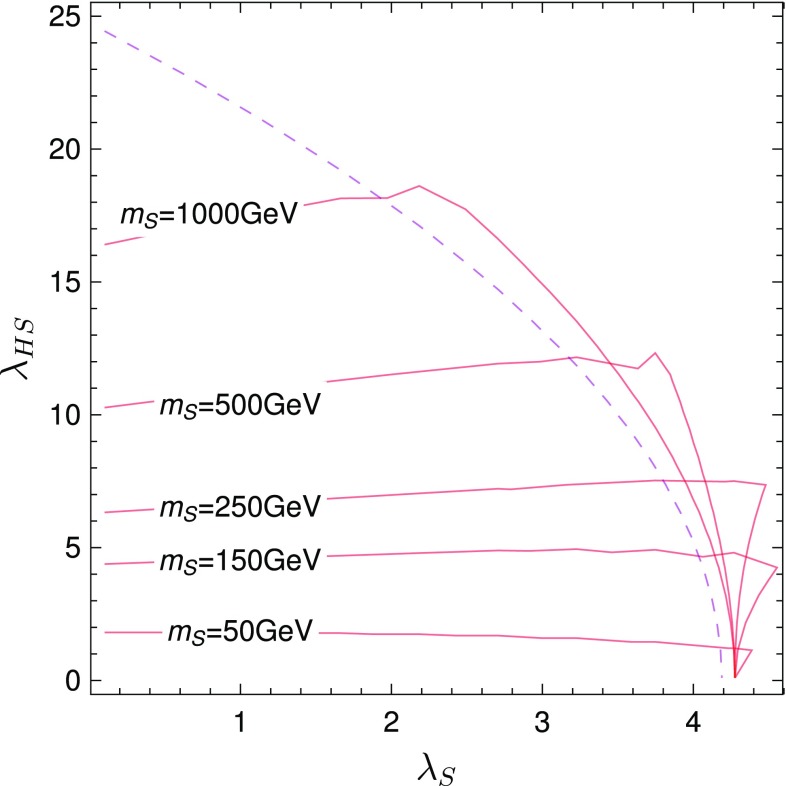
Unitarity constraints for the singlet extended SM. The dashed purple line gives the limit when using only point interactions in the large *s* limit. The red lines give the constraints for the full calculation for different singlet masses

Thus, for $s \sim m_h^2$, this scales as

29 $$\begin{aligned} 16\pi a_0(hS\rightarrow hS)\sim \frac{\lambda _{HS}^2 v^2}{m_h^2} \end{aligned}$$

which can be significantly larger than the limit from point interactions only. This is also confirmed by our numerical calculation with SPheno. In Fig. 3 we compare the limits from including point interaction only with the full calculation in the $(\lambda _S,\lambda _{HS})$ plane for different singlet masses. We see that the unitarity limits on $\lambda _{HS}$ become much stronger for $m_S < m_h$ and small $\lambda _S$. However, even for larger masses a pronounced effect is visible. Even for $m_S=500$ GeV, the limits are stronger by a factor of two.

This brief example demonstrates the importance of going beyond the large *s* approximation when considering unitarity constraints in BSM models. Detailed discussions of these effects in other, phenomenologically more interesting models will be given elsewhere [29, 30].

## Implementation in SARAH

We have extended the Mathematica package by the results and procedures summarised in the previous sections; in particular, the restrictions/assumptions that we apply are described in section 2.3. The user has two possibilities to use the new functionality: (i) during the Mathematica session analytical expressions for specific scattering processes or the full scattering matrix are available; (ii) the necessary routines for a numerical calculation of the unitarity constraints are included in the SPheno output. Below, we give some details how to work with both methods.

### Interactive session in mathematica

#### Commands

In order to obtain analytical expressions for $2\rightarrow 2$ scattering processes or the entire scattering matrix new commands are available in SARAH with version 4.13.0.

1. **Initialisation:** in order to initialise the calculation of the unitarity constraints, one needs to run 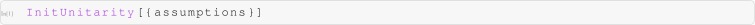 This command calculates all necessary (scalar) vertices. In addition, a list with assumptions can be given which is used to modify the appearance of the vertices. Possible assumptions are:
  Some parameters are neglected, e.g. 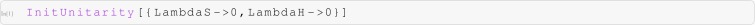

  Mixing between scalars are neglected by replacing rotation matrices with a Kronecker Delta, e.g. 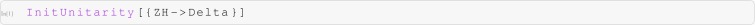

  Some couplings are expressed in terms of other parameters, e.g. 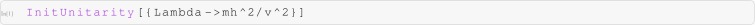

2. **Scattering processes**: once the vertices are initialised, specific scattering processes are obtained via 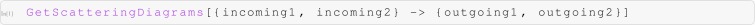 Here, incoming1, incoming2 are the incoming particles and outgoing1, outgoing2 the outgoing ones. One needs to use for these variables the names of fields in SARAH. Optionally, a generation index can also be given.
  No explicit generation indices, e.g. 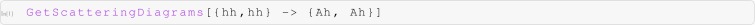 This returns the scattering element for $hh\rightarrow AA$. If the scalar *h* and pseudo-scalar *Ah* appears in several generations in the given model, the indices in1, in2, out1, out2 are used.
  Explicit generation indices, e.g. 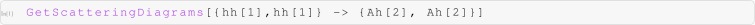 This sets the generation indices of the incoming fields to 1 and of the outgoing fields to 2.
  The result of GetScatteringDiagrams is a function of the couplings and masses in the model. In addition, keywords are introduced to make it possible to trace back the origin of the different terms: *s*, *t* and *u*-channel diagrams as well as point interactions are multiplied with a variable:
  sChan for a *s*-channel diagram
  tChan for a *t*-channel diagram
  uChan for a *u*-channel diagram
  qChan for quartic interactions
  Thus, one can easily remove specific diagrams in order to check their impact by setting the corresponding variables to zero.
3. **Scattering matrix**: the full scattering matrix is return by running 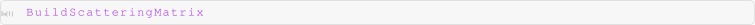 All generation indices of the external fields are explicitly inserted.

#### Example

We show via the example of the SM how the new commands are used in practice. First, SARAH needs to be loaded and the SM be initialised: 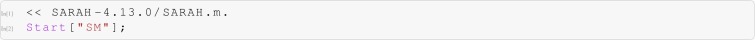 Afterwards, one can start to play with the unitarity constraints. Here, we want to replace the quartic coupling $\lambda$, which is usually used in the vertices, by the Higgs mass. That’s done during the initialisation process of the unitarity constraints. 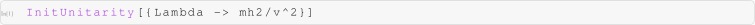 Now, we can take a look at the different scattering processes. The scattering with only the CP even Higgs as external particle is returned by 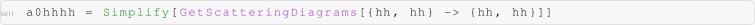 The result is rather lengthy: 
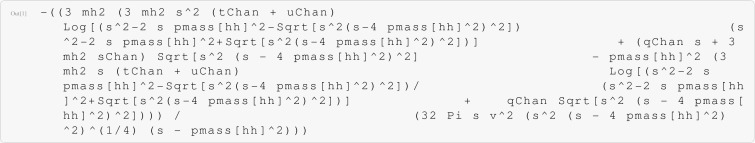
 We can simplify it by introducing a short form of the mass and by setting all filters to 1: 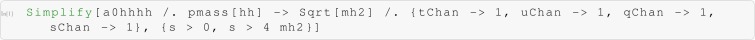 The obtained expression is just the one which was already given by Lee, Quigg and Thacker []: 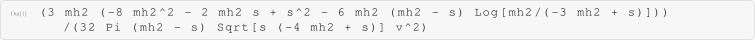 We can now go one step further and calculate the entire scattering matrix. In the case of the SM this is a $10\times 10$ matrix. 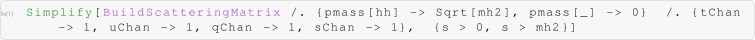 Here, we make here the same assumptions as above. Moreover, we set all masses but the one of the CP even Higgs to zero, i.e. we take the limit $m_Z=m_{H^+}=0$. The outcome is: 
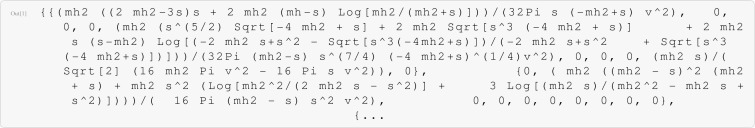
 where we only have shown the first two rows. In order to see the basis in which the matrix is given, one can check 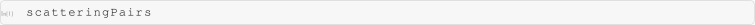 which reads in our case 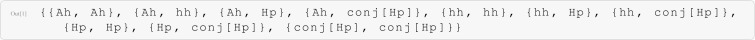


### Including the unitarity constraints in the SPheno Output

While it might be helpful to obtain analytical expressions for some specific channels, in practice a numerical calculation is often more useful. However, Mathematica is not the preferred environment for exhaustive, numerical calculations. Therefore, it was natural to extend the existing SPheno output of SARAH by the new function. So far, SARAH is already producing Fortran source code which can be compiled with SPheno. This provides the possibility to calculate many things for a given model very quickly, e.g. two-loop RGEs, one- and two-loop masses [31–33], flavour and precision constraints [34], two- and three-body decays at tree-level, loop corrections to two-body decays [35], and so on.

#### Generating the Fortran code

The properties of the new spectrum generator based on SPheno are defined within SARAH by using the input file SPheno.m. SPheno.m contains for instance the information about the free input parameters expected from the user, the boundary conditions at different scales, choices for involved scales and several other settings. With SARAH 4.13.0 the following settings are supported: 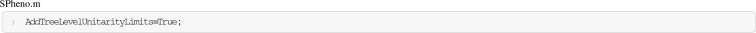 This enables the output of all routines to calculate the tree-level unitarity constraints. By default, this generates the full scattering matrix involving all scalar fields in the model which are colourless. In the case that some particles should not be included, they can be explicitly removed via 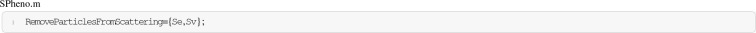 Here, we have for instance decided not to include charged and neutral sleptons in the case of a supersymmetric model. Once SPheno.m for a given model has been edited, one can proceed as usual to obtain the source code and compile:

1. Run 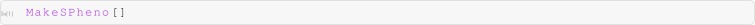 to obtain the source code
2. Copy the code to a new SPheno sub-directory 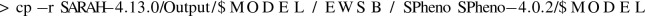

3. Compile the code 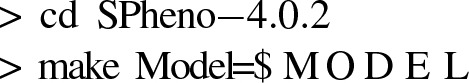

4. Run SPheno 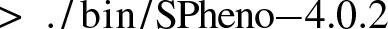

For the last step, a Les Houches input file must be provided which includes the numerical values for the input parameters as well as settings for SPheno.

#### Configuring the unitarity calculations

If the unitarity constraints are turned on the in the SPheno output, several new settings in the Les Houches input file are available: 
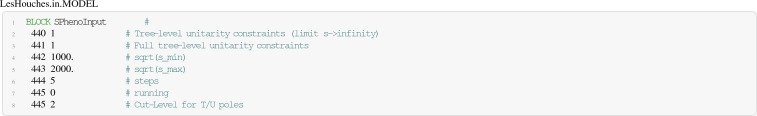

- 440 : the tree-level unitarity constraints in the limit of large $\sqrt{s}$ can be turned on/off. Those include only the point interactions
- 441 : the full tree-level calculations including propagator diagrams can be turned on/off.
- 442 : the minimal scattering energy $\sqrt{s_\mathrm{min}}$ is set
- 443 : the maximal scattering energy $\sqrt{s_\mathrm{max}}$ is set
- 444 : the number of steps in which SPheno should vary the scattering energy between $\sqrt{s_\mathrm{min}}$ and $\sqrt{s_\mathrm{max}}$ is set. SPheno will store the maximal eigenvalue. For positive values, a linear distribution is used, for negative values a logarithmic one.
- 445 : RGE running can be included to give an estimate of the higher order corrections
- 446 : How shall *t* and *u*-channel poles be treated:
  - 0 : no cut at all
  - 1 : only the matrix element with a potential pole is dropped
  - 2 : partial diagonalisation
  - 3 : entire irreducible sub-matrix is dropped
- 447 : The condition to cut out *s*-channel poles

#### The SPheno output

If the unitarity calculations are switched on, the two new blocks appear in the spectrum file written by SPheno: 
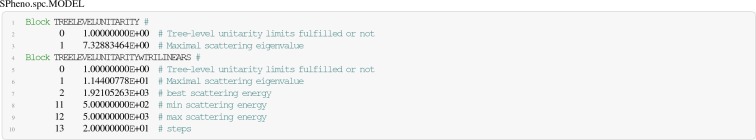
 Thus, SPheno gives two results for the unitarity constrains:

1. TREELEVELUNITARITY: this block contains the old calculation using only point interactions and the large *s* limit
2. TREELEVELUNITARITYwTRILINEARS: this block gives the result for finite *s* including also propagator diagrams

Both blocks contain the following two elements:

- 0 : this is overall result and shows if the point is ruled out (0) or not (1) by the unitarity constraints. The condition for this is that the maximal eigenvalue of the scattering matrix is smaller than 1 / 2.
- 1 : this entry contains the value of the maximal eigenvalue

In addition, the block for the *s*-dependent scattering shows:

- 2 : what is the value for $\sqrt{s}$ at which the scattering is maximised
- 11–13 : this repeats the input for $\sqrt{s_\mathrm{min}}$, $\sqrt{s_\mathrm{max}}$ and the number of steps.

## Summary

We have presented an extension of the Mathematica package SARAH to calculate unitarity constraints in BSM models. It is now possible to obtain predictions for the maximal element of the scattering matrix in a wide range of models without making use of the large *s* approximation. We have provided generic expressions for the calculations, along with pedagogical derivations, and clarified some technical issues concerning additional gauge bosons and the choice of gauge. We have briefly shown the importance of these improved constraints in the example of the real singlet extended SM. More detailed discussions of the effects of the new constraints in doublet and triplet extensions will be given elsewhere [29, 30].

## A: Derivation of the partial wave unitarity constraint

In this appendix we will present an elementary derivation of unitarity constraints, retaining finite momentum factors that are less widely known (and absent from e.g. [5]).

First, we define the *S*-matrix in terms of the interaction matrix *T* as $S=1+iT$. Then in terms of matrix elements of scattering from (multiparticle) states *a* with a set of momentum $\{p\}$ to a set of states *b* with a set of momenta $\{k\}$ we have

A.1 $$\begin{aligned} T_{ba} \equiv \,&{}_\mathrm{out}\langle \{k,b \} |iT| \{p,a\} \rangle _\mathrm{in}\nonumber \\ \equiv \,&i\mathcal {M} ( \{p,a\} \rightarrow \{k,b\}) (2\pi )^4 \delta ^4 (\{k\} - \{p\}) \nonumber \\ \equiv \,&i \mathcal {M}_{ba}(2\pi )^4 \delta ^4 (\{k\} - \{p\}), \end{aligned}$$

and so

A.2 $$\begin{aligned} \mathcal {M}^\dagger ( \{k,b\} \rightarrow \{p,a\}) =&\mathcal {M}^* ( \{p,a\} \rightarrow \{k,b\}). \end{aligned}$$

Now *S* must be a unitary matrix, and so the the constraints from unitarity come from

A.3 $$\begin{aligned} S S^\dagger = 1 \longrightarrow T^\dagger T + i(T-T^\dagger ) = T T^\dagger + i(T-T^\dagger ) = 0. \end{aligned}$$

Then we insert a complete set of states to evaluate $T^\dagger T$:

A.4 $$\begin{aligned}&\langle \{k,b \} |T^\dagger T| \{p,a\} \rangle \nonumber \\&\quad = \sum _{n} d \varPi _n \langle \{k,b \} |T^\dagger | \{q_n, c_n\} \rangle \langle \{q_n, c_n\} | T| \{p,a\} \rangle . \end{aligned}$$

Now specialising to the case of $2\rightarrow 2$ scattering, we can rewrite the equation as

A.5 $$\begin{aligned}&- i (\mathcal {M}_{ba}^{2 \rightarrow 2} - (\mathcal {M}_{ba}^{2 \rightarrow 2})^\dagger ) \nonumber \\&\quad = \sum _c \frac{1}{2^{\delta _c}}\frac{|\mathbf {p}_c|}{16\pi ^2 \sqrt{s}} \int d\varOmega \mathcal {M}_{ca}^{2 \rightarrow 2} \overline{\mathcal {M}}_{cb}^{2 \rightarrow 2}\nonumber \\&\qquad + \underbrace{\sum _{n>2} d \varPi _n d\varOmega \mathcal {M}_{ca}^{2 \rightarrow n} \overline{\mathcal {M}}_{cb}^{2 \rightarrow n}}_{\ge 0}. \end{aligned}$$

Here $\delta _c=0$ if the particles in *c* are not identical, and 1 if they are identical, to allow us to keep the same phase space region of integration (otherwise we double count), and $\mathbf {p}_c$ is the three-momentum in the centre of mass frame for the pair *c*.

Now for the partial wave analysis, we define the three-vectors vectors $\mathbf {p}_a, \mathbf {k}_b, \mathbf {p}_c$ to lie along the unit vectors

A.6 $$\begin{aligned} \hat{k}_a&= (1,0,0) \nonumber \\ \hat{k}_b&= (z_b, \sin \theta _b, 0) \nonumber \\ \hat{k}_c&= (z_c, \sin \theta _c \cos \phi _c, \sin \theta _c \sin \phi _c), \end{aligned}$$

where

$$\begin{aligned} z_b \equiv \cos \theta _b, \qquad z_c \equiv \cos \theta _c. \end{aligned}$$

We decompose the matrices into partial waves:

A.7 $$\begin{aligned} \mathcal {M}_{ca} =\,&16 \pi \sum (2J + 1) P_J (z_c) \hat{a}_J(s) \nonumber \\ \mathcal {M}_{cb} =\,&16 \pi \sum (2J + 1) P_J (\hat{k}_b \cdot \hat{k}_c) \hat{a}_J(s) , \end{aligned}$$

where $P_J$ are the Legendre polynomials, satisfying

A.8 $$\begin{aligned} \int _{-1}^1 dz P_J(z) P_{J'} (z) =&\frac{2}{2J+1} \delta _{JJ'}, \qquad P_0 (z) = 1, \end{aligned}$$

to write

A.9 $$\begin{aligned}&-2\pi i(\hat{a}_J - \hat{a}_J^\dagger )^{ba} \nonumber \\&\quad \le \sum _c \frac{2^{-\delta _c}|\mathbf {p}_c|}{\sqrt{s}} (2J' + 1) (2J''+1)\nonumber \\&\qquad \int d\phi _c dz_c dz_b P_J (z_b) P_{J'} (z_c) P_{J''} (\hat{k}_b \cdot \hat{k}_c) \hat{a}_{J'} \hat{a}_{J''}. \end{aligned}$$

Next we require the identity

A.10 $$\begin{aligned} P_{J} (\hat{k}_b \cdot \hat{k}_c) =&\frac{4\pi }{2 J + 1} \sum _{m=-J}^J Y_{Jm} (\theta _b, \phi _b) Y_{Jm}^* (\theta _c,\phi _c) \end{aligned}$$

where the spherical harmonics satisfy

A.11 $$\begin{aligned} Y_{Jm} \propto e^{im\phi } P_J^m (\cos \theta ), \qquad Y_{J0} = \sqrt{\frac{2J +1}{4\pi }} P_J (\cos \theta ) . \end{aligned}$$

In our case we have $\phi _b =0$ so

A.12 $$\begin{aligned}&-2\pi i (\hat{a}_J- \hat{a}_J^\dagger ) \nonumber \\&\quad \le \sum _c \frac{2^{-\delta _c}|\mathbf {p}_c|}{\sqrt{s}}(2J' + 1) (2J''+1) \nonumber \\&\qquad \int d\phi _c dz_c dz_b P_J (z_b) P_{J'} (z_c) \hat{a}^{J'}_{ca} \overline{\hat{a}}^{J''}_{cb} \frac{4\pi }{2 J'' + 1} \nonumber \\&\qquad \times \sum _m e^{im\phi } P_{J''}^m (z_b ) P_{J''}^m (z_c ) \end{aligned}$$

and thus finally

A.13 $$\begin{aligned} - \frac{i}{2} (\hat{a}_J - \hat{a}_J^\dagger )_{ba} \le&\sum _c \frac{2^{-\delta _c}|2\mathbf {p}_c|}{\sqrt{s}} \hat{a}^{J}_{ca} \overline{\hat{a}}^J_{cb}. \end{aligned}$$

This is true for each partial wave separately.

Now we make the definition:

A.14 $$\begin{aligned} a_J^{ba} \equiv&\sqrt{\frac{4 |\mathbf {p}_b|| \mathbf {p}_a|}{2^{\delta _a} 2^{\delta _b} s}} \hat{a}_J^{ba}. \end{aligned}$$

Then we have

A.15 $$\begin{aligned} - \frac{i}{2} (a_J - a_J^\dagger )^{ba} =&a_J^{ca} \overline{a}^{cb}_J = a_J^{cb} \overline{a}^{ca}_J . \end{aligned}$$

Now, since we could have done this in either order, the matrix $a^J_{ba}$ is normal, and thus both it and $a^\dagger$ can be diagonalised with the same unitary matrix, meaning that we can write for the eigenvalues $(a_J^i)$:

A.16 $$\begin{aligned} \mathrm {Im} (a_J^i) \le | a_J^i|^2. \end{aligned}$$

## B: Scattering amplitudes and partial waves from gauge boson propagators

In this appendix we will clarify the fate of scattering amplitudes among scalars where there is a gauge boson propagator. We neglect all contributions to the final amplitude that are proportional to gauge couplings, but if we work away from the Feynman gauge (it may be desirable to define a theory in that way) then the vector propagators have factors of $1/m_V^2$ where $m_V$ is the vector boson mass – which is proportional to the gauge couplings. In which case we would necessarily need to included gauge boson amplitudes as well as the Goldstone bosons.

We write the couplings of a massive gauge boson to real scalars $\phi _i$ as

B.17 $$\begin{aligned} \mathcal {L}\supset - \frac{1}{2} g^{V ij} A_\mu ^V \phi _i \partial ^\mu \phi _j = - \frac{1}{2} \sum _{i > j} g^{V ij} A_\mu ^V (\phi _i \partial ^\mu \phi _j - \phi _j \partial ^\mu \phi _i), \end{aligned}$$

where *V* now becomes an index. Then the matrix elements for scalar processes $\{1,2\} \rightarrow \{3,4\}$ considering only the gauge boson propagators are

B.18 $$\begin{aligned}&\mathcal {M}^{ba} (\mathrm {Vector\ propagators})\nonumber \\&\quad = - g^{V12} g^{V34} \frac{t-u}{s- m_V^2} \nonumber \\&\qquad - g^{V12} g^{V34} \frac{(m_1^2 - m_2^2)(m_3^2 - m_4^2) }{m_V^2} \bigg ( \frac{1}{s - m_V^2} - \frac{1}{s - \xi m_V^2} \bigg ) \nonumber \\&\qquad + \bigg ( (2 \leftrightarrow 3),\ (s \leftrightarrow t) \bigg ) + \bigg ( (2 \leftrightarrow 4),\ (s \leftrightarrow u) \bigg ). \end{aligned}$$

To these, we should add the contributions from the Goldstone bosons. In [35] it was shown that, for scalars coupling to the corresponding golstone boson with the same index *V*

B.19 $$\begin{aligned} \mathcal {L}\supset - \frac{1}{2} \kappa ^{Vij} G_V \phi _i \phi _j \end{aligned}$$

that the couplings are related by

B.20 $$\begin{aligned} \kappa ^{Vij} = \frac{m_i^2 - m_j^2 }{m_V} g^{Vij}. \end{aligned}$$

Hence when we add the contribution from the Goldstone bosons, we just obtain

B.21 $$\begin{aligned}&\mathcal {M}^{ba} (\mathrm {Vector\ propagators+\ Goldstones})\nonumber \\&\quad = - g^{V12} g^{V34} \frac{t-u}{s- m_V^2} - \kappa ^{V12} \kappa ^{V34} \frac{1}{s - m_V^2} \nonumber \\&\qquad + \bigg ( (2 \leftrightarrow 3),\ (s \leftrightarrow t) \bigg ) + \bigg ( (2 \leftrightarrow 4),\ (s \leftrightarrow u) \bigg ). \end{aligned}$$

This result is manifestly gauge invariant. Setting the gauge couplings $g^{V12}$ to zero, we have a remaining piece which is just equal to the contribution from the Goldstone bosons in Feynman gauge! However, we should note that in other gauges it is necessary to include the gauge boson propagators; for example, if we work in unitary gauge then there are no Goldstone boson propagators!

As an aside, if we want to include the contributions from heavy gauge bosons (i.e. not neglect their couplings), it is simple to perform the angular integrations for these contributions, using:

B.22 $$\begin{aligned}&M^2 \equiv m_1^2 + m_2^2 + m_a^2 + m_b^2 = s+t+u\nonumber \\&\frac{s-u}{t- m_V^2} = \frac{2s-M^2-m_V^2}{t- m_V^2} -1 \end{aligned}$$

B.23 $$\begin{aligned}&\int _{-1}^1 dz \frac{t-u}{s- m_V^2} = \frac{2}{s-m_V^2} \frac{(m_3^2 - m_4^2)(2s - m_1^2 + m_2^2)}{2s} . \end{aligned}$$

We find

B.24 $$\begin{aligned} \varDelta a_{0}&= \frac{1}{16\pi \sqrt{2^{\delta _{12}} 2^{\delta _{34}}}}\nonumber \\&\quad \times \, \bigg \{ \frac{g^{V12} g^{V34}}{s-m_V^2} \sqrt{\frac{4 |\mathbf {p}_1 ||\mathbf {p}_3|}{s}} \frac{(m_3^2 - m_4^2)(2s - m_1^2 + m_2^2)}{2s}\nonumber \\&\quad + g^{V13} g^{V24} \bigg [ (2s - M^2 - m_V^2) f_t(s,m_1^2,m_2^2, m_3^2,m_4^2,m_V^2) - 1\bigg ]\nonumber \\&\quad + g^{V14} g^{V23} \bigg [ (2s - M^2 - m_V^2) f_t(s,m_1^2,m_2^2, m_4^2,m_3^2,m_V^2) - 1\bigg ]\bigg \}. \end{aligned}$$

## C: Scattering Elements in the real singlet extended SM

C.25 $$\begin{aligned}&a_0(hh\rightarrow hh)=-\frac{3 m_h^2 \left( -8 m_h^4-2 m_h^2 s-6 \left( m_h^4-m_h^2 s\right) \log \left( \frac{m_h^2}{s-3 m_h^2}\right) +s^2\right) }{32 \pi v^2 \sqrt{s \left( s-4 m_h^2\right) } \left( s-m_h^2\right) } \end{aligned}$$

C.26 $$\begin{aligned}&a_0(hh\rightarrow SS)= -\frac{\lambda _{HS} \left( 2 \lambda _{HS} v^2 \left( s-m_h^2\right) \log \left( \frac{-\sqrt{\left( s-4 m_h^2\right) \left( s-4 m_S^2\right) }-2 m_h^2+s}{\sqrt{\left( s-4 m_h^2\right) \left( s-4 m_S^2\right) }-2 m_h^2+s}\right) +\left( 2 m_h^2+s\right) \sqrt{\left( s-4 m_h^2\right) \left( s-4 m_S^2\right) }\right) }{32 \pi \left( s-m_h^2\right) \root 4 \of {s^2 \left( s-4 m_h^2\right) \left( s-4 m_S^2\right) }} \end{aligned}$$

C.27 $$\begin{aligned}&a_0(hS\rightarrow hS) =-\frac{\lambda _{HS}}{16 \pi s \left( s-m_S^2\right) \sqrt{m_h^4-2 m_h^2 \left( m_S^2+s\right) +\left( m_S^2-s\right) ^2}} \nonumber \\&\times \Bigg [{-}\left( m_h^4-2 m_h^2 \left( m_S^2+s\right) {+}\left( m_S^2-s\right) ^2\right) \left( -\lambda _{HS} v^2{+}m_S^2-s\right) +\lambda _{HS} s v^2 \left( s-m_S^2\right) \log \left( \frac{m_h^4-2 m_h^2 m_S^2+m_S^4-m_S^2 s}{s \left( 2 m_h^2+m_S^2-s\right) }\right) \nonumber \\&+3 m_h^2 s \left( s-m_S^2\right) \log \left( \frac{m_h^2 s}{m_h^4-m_h^2 \left( 2 m_S^2+s\right) +\left( m_S^2-s\right) ^2}\right) \Bigg ] \end{aligned}$$

C.28 $$\begin{aligned}&a_0(SS\rightarrow SS)= \frac{\left( 4 m_S^2-s\right) \left( \lambda _{HS}^2 v^2-12 \lambda _{S} \left( m_h^2-s\right) \right) +2 \lambda _{HS}^2 v^2 \left( m_h^2-s\right) \log \left( \frac{m_h^2}{m_h^2-4 m_S^2+s}\right) }{32 \pi \left( s-m_h^2\right) \sqrt{s \left( s-4 m_S^2\right) }} \end{aligned}$$

C.29 $$\begin{aligned}&a_0(hh\rightarrow ZZ) = \frac{2 \left( m_h^6-m_h^4 s\right) \log \left( \frac{-\sqrt{\left( s-4 m_h^2\right) \left( s-4 m_Z^2\right) }-2 m_h^2+s}{\sqrt{\left( s-4 m_h^2\right) \left( s-4 m_Z^2\right) }-2 m_h^2+s}\right) -m_h^2 \left( 2 m_h^2+s\right) \sqrt{\left( s-4 m_h^2\right) \left( s-4 m_Z^2\right) }}{32 \pi v^2 \left( s-m_h^2\right) \root 4 \of {s^2 \left( s-4 m_h^2\right) \left( s-4 m_Z^2\right) }} \end{aligned}$$

C.30 $$\begin{aligned}&a_0(hZ\rightarrow hZ) = \frac{m_h^2}{16 \pi s v^2 \left( s-m_Z^2\right) \sqrt{m_h^4-2 m_h^2 \left( m_Z^2+s\right) +\left( m_Z^2-s\right) ^2}} \Bigg [m_h^2 s \left( m_Z^2-s\right) \left( \log \left( \frac{\left( m_h^2-m_Z^2\right) ^2-m_Z^2 s}{s \left( 2 m_h^2+m_Z^2-s\right) }\right) \right. \nonumber \\&\left. +3 \log \left( \frac{m_h^2 s}{m_h^4-m_h^2 \left( 2 m_Z^2+s\right) +\left( m_Z^2-s\right) ^2}\right) \right) -\left( (m_h-m_Z)^2-s\right) \left( (m_h+m_Z)^2-s\right) \left( m_h^2-m_Z^2+s\right) \Bigg ] \end{aligned}$$

C.31 $$\begin{aligned}&a_0(SS\rightarrow ZZ) = \frac{\lambda _{HS} \sqrt{s} \root 4 \of {\left( s-4 m_S^2\right) \left( s-4 m_Z^2\right) }}{32 \pi m_h^2-32 \pi s} \end{aligned}$$

C.32 $$\begin{aligned}&a_0(ZZ\rightarrow ZZ) = \frac{m_h^2 \left( \left( 2 m_h^2-3 s\right) \left( s-4 m_Z^2\right) +2 \left( m_h^4-m_h^2 s\right) \log \left( \frac{m_h^2}{m_h^2-4 m_Z^2+s}\right) \right) }{32 \pi v^2 \left( s-m_h^2\right) \sqrt{s \left( s-4 m_Z^2\right) }} \end{aligned}$$

C.33 $$\begin{aligned}&a_0(hh\rightarrow WW) = \sqrt{2}(a_0(hh\rightarrow ZZ) | m_Z \rightarrow m_W)\end{aligned}$$

C.34 $$\begin{aligned}&a_0(hW\rightarrow hW) = (a_0(hZ\rightarrow hZ) | m_Z \rightarrow m_W)\end{aligned}$$

C.35 $$\begin{aligned}&a_0(SS\rightarrow WW) = \sqrt{2}(a_0(SS\rightarrow ZZ) | m_Z \rightarrow m_W)\end{aligned}$$

C.36 $$\begin{aligned}&a_0(WW\rightarrow WW)= \frac{m_h^2 \left( \left( m_h^2-2 s\right) \left( s-4 m_W^2\right) +\left( m_h^4-m_h^2 s\right) \log \left( \frac{m_h^2}{m_h^2-4 m_W^2+s}\right) \right) }{16 \pi v^2 \left( s-m_h^2\right) \sqrt{s \left( s-4 m_W^2\right) }} \end{aligned}$$
